# Mr. Hill, on Inoculation

**Published:** 1805-07-01

**Authors:** Samuel Hill

**Affiliations:** Queen Street, Portsea


### *To the Editors of the Medical and Physical Journal.*

GENTLEMEN,

IF you think the following fact, shewing the strong anti-variolous power of the vaccine fluid of sufficient importance, you will oblige me by giving it a place in your very useful Journal. I am aware that many of your readers have seen similar instances of protection afforded against variolous contagion, by vacciolation; and I feel regret at occupying your pages, which may be more usefully filled, than by attempts to prove, what no one ought to doubt; but,

Bonum quo communius eo melius.

On which principle I take the liberty of sending you a statement of the case, as it occurred.

A poor woman, named Sibly, who lives at No. 20, Love Lane, Portsea, brought two children to me on the 29th ult. to be vacciolated; I desired her to bring them next morning, when I expected a child to be brought me for inspection, who had been inoculated eight days, and from whom I could do it with recent fluid. She called accordingly (30th) but brought only James, aged two years, and informed me that Mary, aged seven years, had been taken very ill, the evening before, and still continued so, for which reason she declined having her vacciolated, until the fever should be removed.

May 30th. James was this day vacciolated, and was brought to me on the 2d of June, when I was satisfied that it had taken. The mother informed me this day that Mary had the small-pox out very thick, was still very ill, and the head much affected. I desired her to let the children remain together; she said, she had only one bed-room, and consequently could not separate them.

June 5. The vaccine vesicles on James were proceeding favourably, and I was desired to see Mary. She had the confluent small-pox, very full, and was so ill as to require two attendants day and night.

9th. The pocks were filled with an immense quantity of matter; the room was highly offensive, and she moaned piteously; was very restless, except when under the influence of anodynes, which were absolutely necessary to keep her quiet.

11th.

11th. The pocks on her face began to turn, and successively those on the body and extremities. She now became so composed, that anodynes were discontinued.

On James, the vaccine vesicles proceeded in the most regular manner through all their stages, forming the areola, and this day (13th inst.) the horny eschar.

It was highly gratifying to me and the parents of the children, to see James run about the room, jump upon the bed, at the same time eating his hard pudding; it certainly formed a most pleasing contrast to the situation and suffering of the other poor child. If she had been vacciolated (which I would have done, had the mother consented) when her brother was, I think, that, if it had not entirely prevented the variolous disease, it most likely would have rendered it much more mild, by producing only a few distinct pustules, which would have lessened her suffering, the anxiety of her parents, the consequent expence, and subsequent deformity. Her eyes were closed several days, but the sight is not injured.

Two medical friends of Portsea saw the children, and expressed themselves quite satisfied, that the vacciolated child was perfectly secure against variolous infection.

In a book published by, and which I had the honor to receive from, the Royal Jennerian Society,* it is mentioned p. 42, among the probable causes of spurious pustule, "The genuine vesicle having been destroyed at an early stage, and the regular progress of the disease thus interrupted." Since I read this passage I have always vacciolated, *in at least two places;* by so doing, I can always obtain an ample supply of the vaccine principle, both for my friends and myself, without breaking down *all the vesicles;*† indeed, I always endeavour to let one go on to perfection, without interrupting its progress, to ensure the safety of my patient. If children render themselves insecure by scratching the vesicle, which I believe; may not breaking it down to take the vaccine fluid have the same effect? In taking the fluid perhaps we do not destroy *all the cells,*‡ the subject will remain of course secure; but in

* Entitled, Address of the Royal Jennerian Society for the Extermination of the Small-pox, &c.

† I am led to make these remarks by reading a passage in Dr. Walker's letter in your Journal for May, p. 456, on breaking down the vaccine vesicle, and Mr. Dunning's comment on the same, in the Journal for June, p. 542.

‡ Suppose we have only one vesicle.

 the the scratching of children, which is extremely difficult to prevent, the vesicle is nineteen times out of twenty completely destroyed; in which cases, I always revacciolate.

*Queen Street, Portsea.* I am, &c.

*June* 13, 1804. SAMUEL HILL.

